# Economic evaluation of policy options for dialysis in end-stage renal disease patients under the universal health coverage in Indonesia

**DOI:** 10.1371/journal.pone.0177436

**Published:** 2017-05-18

**Authors:** Afiatin, Levina Chandra Khoe, Erna Kristin, Lusiana Siti Masytoh, Eva Herlinawaty, Pitsaphun Werayingyong, Mardiati Nadjib, Sudigdo Sastroasmoro, Yot Teerawattananon

**Affiliations:** 1 Department of Internal Medicine, Faculty of Medicine, Universitas Padjajaran, Bandung, Indonesia; 2 Department of Community Medicine, Faculty of Medicine, Universitas Indonesia, Jakarta, Indonesia; 3 Department of Pharmacology and Therapy, Faculty of Medicine, Universitas Gadjah Mada, Jogjakarta, Indonesia; 4 Centre for Health Financing and Security, Ministry of Health, Jakarta, Indonesia; 5 Health Intervention and Technology Assessment Program (HITAP), Ministry of Public Health, Bangkok, Thailand; 6 Indonesian Health Technology Assessment Committee, Jakarta, Indonesia; Postgraduate Medical Institute, INDIA

## Abstract

**Objectives:**

This study aims to assess the value for money and budget impact of offering hemodialysis (HD) as a first-line treatment, or the HD-first policy, and the peritoneal dialysis (PD) first policy compared to a supportive care option in patients with end-stage renal disease (ESRD) in Indonesia.

**Methods:**

A Markov model-based economic evaluation was performed using local and international data to quantify the potential costs and health-related outcomes in terms of life years (LYs) and quality-adjusted life years (QALYs). Three policy options were compared, i.e., the PD-first policy, HD-first policy, and supportive care.

**Results:**

The PD-first policy for ESRD patients resulted in 5.93 life years, equal to the HD-first policy, with a slightly higher QALY gained (4.40 vs 4.34). The total lifetime cost for a patient under the PD-first policy is around 700 million IDR, which is lower than the cost under the HD-first policy, i.e. 735 million IDR per patient. Compared to supportive care, the incremental cost-effectiveness ratio of the PD-first policy is 193 million IDR per QALY, while the HD-first policy resulted in 207 million IDR per QALY. Budget impact analysis indicated that the required budget for the PD-first policy is 43 trillion IDR for 53% coverage and 75 trillion IDR for 100% coverage in five years, which is less than the HD-first policy, i.e. 88 trillion IDR and 166 trillion IDR.

**Conclusions:**

The PD-first policy was found to be more cost-effective compared to the HD-first policy. Budget impact analysis provided evidence on the enormous financial burden for the country if the current practice, where HD dominates PD, continues for the next five years.

## Introduction

End-stage renal disease (ESRD) is a major global burden of disease, affecting not only mortality and morbidity, but also the economic condition of a country. The growing concern about ESRD is driven by population ageing and an increasing prevalence of non-communicable diseases.[1–3] Renal replacement therapy (RRT), i.e. kidney transplantation, hemodialysis (HD), peritoneal dialysis (PD), is essential for the treatment of ESRD patients. Without dialysis, the prognosis of ESRD patients is varied, between six months to nearly two years.[4–5] In Asia, the number of ESRD patients receiving RRT is projected to double from 2.6 million in 2010 to 5.4 million by 2030.[1] In Indonesia, a nationwide household survey on basic health research showed that 0.2% of the population are at risk of developing ESRD.[6] Kidney transplantation remains the best treatment option for ESRD patients,[7–9] however, the paucity of living organs and poorly accepted cadaveric donors limit patients’ choices to either HD or PD.[10] Moreover, given the annual incidence of 35,000 patients[11] and prevalence of 120,000 patients,[12] kidney transplantation is not a viable option. Indonesian Renal Registry reported HD as the most preferable treatment option, comprising 80% of all ESRD patients, and only 2% for PD.[13]

A premium-based national health insurance scheme, namely *Jaminan Kesehatan Nasional* (JKN), managed by the Health Social Security Institution (BPJS) was launched in 2014. The JKN aims to achieve universal health coverage for an estimated population of 250 million by 2019. All members of JKN would receive a wide range of health services by public providers, as well as private if they opted to join the JKN scheme. However, the members must pay a certain premium depending on their ability and willingness to pay. The implication of the different premium is in terms of ward class. All dialysis treatments are reimbursed under this insurance scheme with a higher reimbursement rate for HD than PD. It is estimated that only 53% of patients have access to dialysis and almost all of them are undertaking HD, although PD is less expensive than HD.[14] With the current dialysis coverage, more than IDR 1.5 trillion (1 USD ≈13,500 USD) was spent in 2014, which is the second largest expense for BPJS.[15] Since the cost of dialysis heavily burdens the healthcare system, various economic evaluations to assess the financial impact of dialysis treatment has been performed in many settings worldwide.[16–21] In many developed countries, HD costs more than PD, while in developing countries, there were some exceptions where PD was reported to be more expensive than HD.[19] Based on health and economic reasons, public health authorities in Thailand and Hong Kong have implemented PD as the initial treatment for ESRD patients and successfully increased the coverage of dialysis treatment.[21,22] They offer financial incentives to both providers and patients to undertake PD as the first-line treatment; detailed information about the programs are described elsewhere.[23]

This study involved conducting a model-based economic evaluation and budget impact analysis on the first choice of dialysis modality, either HD or PD, for ESRD patients. Under the HD-first policy, ESRD patients are provided with HD as initial care followed by PD if complications/switching occur. For the PD-first policy, patients are offered PD as initial care followed by HD if complications/switching occur.[24–26] Supportive care was used as a comparator, although this was not commonly practiced in daily clinical practice, in order to illustrate how many people could access or how many lives could be saved by implementing PD or HD first policy. This study aims to provide scientific evidence for policymakers, showing which treatment strategies should be implemented to cover all ESRD patients and evaluating the value for money of providing HD and PD first policy compared to supportive care.

## Materials and methods

**Fig 1:**
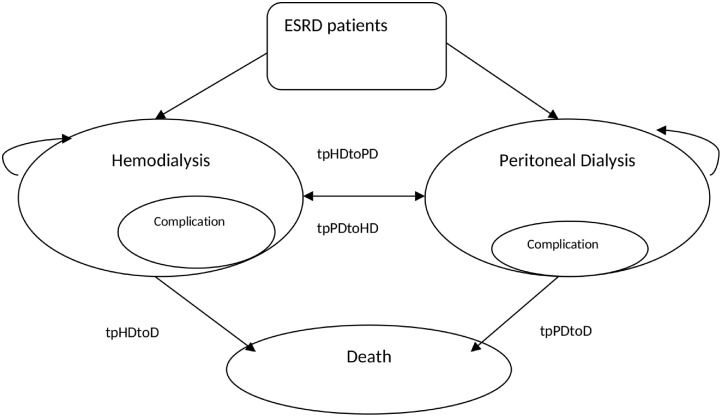
Schematic diagram of Markov model.

A Markov model was developed using Microsoft Excel 2010 to quantify the costs and health outcomes in terms of life years (LY) and quality-adjusted life years (QALY). Three policy options were compared: 1) the HD-first policy; 2) the PD-first policy; and 3) supportive care, defined in terms of fluid restriction, high-dose diuretics, hypertensive drugs, calcium bicarbonate, ferrous sulfate, and blood transfusion provided for ESRD patients who cannot afford dialysis or kidney transplantation (Fig 1). In the model, JKN patients may commence with either PD or HD depending on the policy choice and continue on the same treatment until the next cycle. Patients may experience complications during the treatment and switch to another modality, or may die of either ESRD or non-ESRD causes. The model used a one-year cycle length for health state and one month for complication sub-states. Lifetime horizon was applied in this model. Using this approach, total lifetime cost and health outcomes amongst the three policy options were compared. Societal and healthcare provider perspectives were adopted for this model; therefore, direct medical, direct non-medical and indirect costs were included in the analysis. All costs were presented in the year 2015 and discounted at a rate of 3.0% for both costs and outcomes. The threshold of one GDP, i.e. IDR 43 million per QALY gained, was applied in this study since the threshold value per QALY gained is not yet available in Indonesia.

The parameters we used in this study is compiled in Table 1.

**Table 1:** Means and standard error of input parameters.

Parameters	Mean	SE	Distribution	Data Source
***Survival data***				
Year 1	0.224	0.0018	Beta	IRR
Year 2	0.135	0.0023	Beta	IRR
Year 3	0.122	0.0028	Beta	IRR
Year 4	0.059	0.0033	Beta	IRR
Year 5	0.063	0.0041	Beta	IRR
Year 6	0.146	0.0184	Beta	IRR
***Transitional probabilities***				
Probability of dying among patients with supportive care	0.405	0.159	Beta	Expert opinion
Probability of having peritonitis among PD patients	0.252	0.502	Gamma	[26]
Probability of having vascular access related complication among HD patients	0.041	0.040	Gamma	[13]
Probability of switching from HD to PD	0.111	0.072	Beta	[23,24]
Probability of switching from PD to HD	0.350	0.104	Beta	[23,24]
***Direct medical costs*** (S1 File)				
Cost of supportive care	444,400	444,400	Gamma	INA CBGs*
Cost of initial PD	13,724,975	141,669	Gamma	Billing
Cost of initial HD	13,822,775	2,102,552	Gamma	Billing
Annual maintenance cost for PD	88,926,820	2,978,939	Gamma	Billing
Annual maintenance cost for HD	110,402,541	2,545,667	Gamma	Billing
Annual cost of treating PD complications	8,207,800	3,575,200	Gamma	Billing
Annual cost of treating HD complications	12,924,542	6,125,203	Gamma	Billing
***Direct non-medical costs*, *e*.*g*. *travel*, *food***				
Lifetime cost paid by household with supportive care	274,881	274,881	Gamma	Questionnaire
Annual cost paid by household with PD	1,554,789	249,450	Gamma	Questionnaire
Annual cost paid by household with HD	9,004,780	793,556	Gamma	Questionnaire
***Indirect non-medical costs*, *e*.*g*. *income loss*** (S2 File)				
Annual cost paid by household with PD	1,743,783	1,302,865	Gamma	Questionnaire
Annual cost paid by household with HD	3,156,480	8,061,043	Gamma	Questionnaire
***Utility***				
Utility for PD without complication	0.82	0.03	Beta	Questionnaire
Utility for HD without complication	0.70	0.04	Beta	Questionnaire
Utility for PD with complication	0.31	0.09	Beta	Questionnaire
Utility for HD with complication	0.37	0.11	Beta	Questionnaire

*INA CBGs tariff set by government according to the diagnosis, type of hospital, and regions.

The data were obtained primarily from hospital billing and medical records for a one-year period (1 June 2014 to 30 June 2015). We also compared the direct medical cost data with the reimbursement tariff stated in the Regulation of Health Minister No 59, year 2014 [27]. Direct non-medical costs, indirect costs, and utilities were derived from interviews with patients using a questionnaire. For direct non-medical costs, all subjects were asked about their travel, food, and accommodation expenses when they visited hospitals. The direct non-medical costs pertained to the costs expended by patients and caregivers, while the indirect costs referred to the income loss of the caregiver in a year. The questionnaire on costing consisted of 41 questions on demographics, and direct non-medical and indirect costs. The questions had been tested among 30 patients in a dialysis center in Jogjakarta and validated by statisticians.

A total of 104 patients were recruited from three hospitals, with 52 patients for PD and HD each. (S3 File) The inclusion criteria for the study population were ESRD patients treated with hemodialysis (HD) or peritoneal dialysis (PD), adults (older than 18 years old), had received a minimum of six-month therapy with either HD or PD, and had no contraindications of both modalities (including the incapability of doing PD at home). Patients who were discontinue dialysis therapy within three months or had combination therapy of both HD and PD were excluded from the study. The characteristics of HD and PD patients were matched by data on age, gender, therapy duration, and co-morbidity.

Utility values were obtained from the EuroQoL EQ-5D-3L Indonesian version. Patients were asked about their current condition and their condition during complication (if any). Without complication, the utility data of PD patients and HD patients, respectively, was 0.82 and 0.70. Utility data for PD and HD patients with complication were 0.31 and 0.37 respectively. The utility scores of PD and HD patients were calculated using Thailand’s value set.[28]

A probabilistic sensitivity analysis using a Monte Carlo simulation was conducted in this study. All input parameters were assigned a probability distribution to reflect a possible range of its values. The process was repeated for 1,000 simulations. Each simulation provided one value of cost-effectiveness. The average value of the probabilistic sensitivity analysis was presented as the ICER value and the cost-effectiveness acceptability curve.

Budget impact analysis was carried out to assess the financial impact of providing PD-first policy or HD-first policy in the perspective of the healthcare provider. The budget was estimated for a five-year time horizon. The input data included the prevalence, incidence, and coverage of dialysis treatment. The prevalence was obtained from BPJS claim data in the year 2014,[12] whereas the incidence was taken from the Indonesian Renal Registry Report in 2014.[11] A prevalence of population size of 63,818 patients and incidence of 17,913 patients were modelled in this study. Two scenarios of coverage (53% and 100%) were examined in the budget impact analysis. The coverage data was calculated from the number of ESRD patient who accessed dialysis in the year 2014 divided by the expected total number of ESRD patient.

## Results

The supportive care policy option resulted in 0.21 life years saved or 0.076 QALY. Without discounting the health outcomes and using equal survival values, both the PD- and HD-first policy options resulted in 5.93 life years saved. However, the QALYs gained for the PD-first policy option was slightly higher than the HD-first policy, i.e. 4.40 vs 4.34 QALYs because PD patients experienced a higher quality of life compared to HD patients.

Using the societal perspective, the total cost of supportive care was 1.7 million IDR, while the PD-first policy was 696.6 million IDR and the HD-first was 735.4 million IDR. In the provider perspective, the total cost of supportive care was 1.1 million IDR, the PD-first policy was 674.1 million IDR, and the HD-first was 672.4 million IDR. From both perspectives, the HD-first policy had the highest costs among the three options and indicated a large burden borne by households, whereas the supportive care was the least costly options.

**Fig 2:**
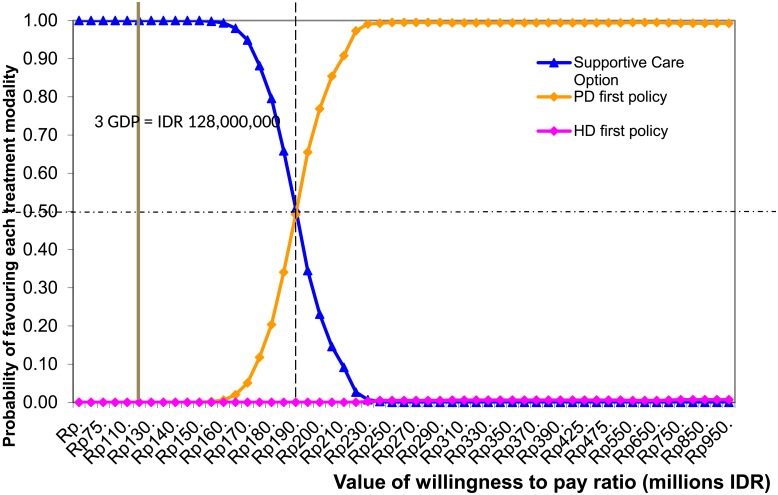
Cost effectiveness acceptability curves.

The incremental cost-effectiveness ratio (ICER) of providing the PD-first policy was 193.2 million IDR per QALY gained, which was lower than the HD-first policy, with ICER value of 207.4 million IDR per QALY gained. The cost-effectiveness acceptability curve (Fig 2) showed the probability of favoring each option dependent on the level of willingness to pay. At willingness to pay of 43 million IDR, supportive care was the best option. The PD-first policy was the best option if the willingness to pay was more than 190 million IDR. Irrespective of the willingness to pay, the HD-first policy was not cost-effective because it provided a higher cost but lower QALY than the PD-first policy.

The budget impact analysis indicated that introducing PD or HD as the first choice would require 6.7 trillion IDR and 8.0 trillion IDR in the first year, and 39.4 trillion IDR and 87.7 trillion IDR over five years, respectively. If increasing the access to dialysis treatment to 100 percent, it would require 74.5 trillion IDR and 165.7 trillion IDR over five years to provide PD-first and HD-first policy, respectively.

## Discussion

The results of this study indicated that neither the PD- nor the HD-first policy provides good value for money when the ceiling ratio is similar to one GDP or 43 million IDR. However, there is currently no agreed threshold for adopting health technologies in Indonesia. If the government increases the willingness to pay to or higher than 190 million IDR, which is more than three times GDP, the PD-first policy would be the optimal decision since it dominated the HD-first policy at any value of the threshold. These findings are consistent with prior studies investigating similar topics.[16,17,21]

Using societal perspective, this study assessed the household burden due to dialysis treatment. HD households spent higher on transportation costs than PD since they require more visits to the hospitals (9 million vs 2 million IDR, respectively). The current study used urban settings with the average transportation time from a patient’s house to the hospital of less than one hour, whereas in rural settings patients may need several hours just to travel to the clinic. Since Indonesia has a wide geographic variation, PD should be considered as a superior treatment choice to HD since it requires less traveling costs for households and, for healthcare providers, diminishes the need to build new facilities, buy expensive machines, and employ more highly trained staff. Establishing new HD centers in remote areas with a low population will increase the cost of HD in future. However, the current cost of PD is considerably high, especially for PD solution, which requires distribution costs and special training for medical staff. Additionally, there are some issues related with the limitation of supplier for both modalities in Indonesia. Supplier for HD is still considered sufficient at the present (53% of dialysis coverage), but may not be enough to cover the future needs. While supplier for PD is currently insufficient.

Patients’ quality of life were measured using EQ-5D-3L, which was well-accepted in prior studies.[29] HD patients had generally lower scores than PD for all five dimensions in EQ-5D, i.e. mobility, self-care, usual activity, pain/discomfort, and anxiety/depression (0.78 vs 0.85 respectively). The results were in accordance with Thailand’s study,[21] but had some discrepancies with other studies that showed similar or severe impairment in PD patients compared to HD.[28,29]

The budget impact analysis revealed that the PD-first policy carries lower financial burden to the payer compared to HD first. Given the current coverage was only 53% and assuming all patients will have access to dialysis by 2019, considerable savings could be made for the payer if PD was offered as the initial treatment for ESRD patients. It should be emphasized that the term “PD first” does not eliminate HD or other modality options, and vice versa. It is acknowledged that a patient’s choice on dialysis modality is influenced by age, physical condition, comorbidities and lifestyle.[30,31] Therefore, in this study, we included the probability of switching in the calculation of the Markov model. Note that in this model, both HD and PD would need to be available, but applied at different coverage levels.

The key strength of the present study is that it offers the first evidence using model-based economic evaluation and budget impact analysis on the dialysis modality choices for ESRD patients in Indonesia. Furthermore, the model and input parameter was validated at numerous panel meetings with well-respected nephrologists, academicians, and health economic experts.

The limitation in our study was the lack of PD survival data. The follow up period of survival data was 6 years and the study is still ongoing. At that point in time, 44.3% of HD patients were still alive. There was no local data on the survival of PD patients due to a very small number of PD patients in Indonesia, compared to HD. Moreover, studies showed no significant differences in terms of survival rate between PD and HD. Therefore, we used similar survival data for PD patients.

This study was performed using billings from three hospitals with different classes, which applied different tariffs. Hence, the results from economic evaluation have little generalizability across different regions in Indonesia. Further studies investigating PD and HD on a bigger scale to include different type of hospitals and regions are needed to understand a complete picture of the dialysis treatment in Indonesia.

Additionally, the utility value for patients with supportive care is assumed equal to the utility for dialysis with complications. Nevertheless, the use of this value was accepted since the incidence of having no dialysis is very rare and it was also applied in another study.[21] The value set used in this study was taken from Thailand since local data was not available during this study.

## Conclusions

Based on this study, the PD-first policy was recommended as the best option if the willingness to pay increases to more than 190 million IDR. Moreover, the necessary budget to implement PD as the first treatment option was less than offering HD as the first option in five years’ time. Nevertheless, in order to establish the PD-first policy, we should acknowledge the current problems in increasing PD coverage. More effort and commitment from the government and healthcare professionals should be made to ensure the successful implementation of the PD-first policy.

## Supporting information

S1 File Direct cost. (PDF)

S2 File Indirect cost. (PDF)

S3 File Demographic data. (PDF)
