# Supplementary material for: Economic evaluation of policy options for dialysis in end-stage renal disease patients under the universal health coverage in Indonesia
Source: PLoS One. 2017 May 18;12(5):e0177436. doi: 10.1371/journal.pone.0177436 (PMC5436694; doi:10.1371/journal.pone.0177436)
Supplement: S1 File — (PDF) [file pone.0177436.s001.pdf]

CAPD

Routime Costs included routine CAPD dialysis and other comorbidities (Both Outpatient and In-patient)

No	Direct Med Cost for Routine CAPD in a year	Catheter	Training	Initial PD	PD Complication
1	132,958,763				
2	118,126,954				
3	114,375,842				
4	133,806,878				
5	89,445,268				
6	114,824,843				
7	108,826,309				
8	125,753,421				9,858,000
9	89,949,591				
10	95,153,871				
11	90,482,174				
12	109,874,770				
13	103,915,923				
14	70,896,821				
15	92,795,250				
16	92,795,250				
17	97,669,508				
18	141,380,459				
19	97,110,648				
20	109,386,648				
21	125,302,509				
22	118,085,702				
23	72,992,719				
24	73,775,582				
25	73,700,208				
26	73,059,150				
27	73,551,892				
28	73,846,294	9,790,900	1,729,300	11,520,200	
29	74,371,050				
30	73,160,833				
31	77,362,045	9,790,900	1,668,100	11,459,000	
32	72,442,876	9,790,900	1,287,600	11,078,500	2,707,600
33	72,925,713				
34	73,719,784				
35	73,643,277	9,790,900	1,151,300	10,942,200	
36	72,678,933				

37	73,907,320
38	73,907,272
39	72,922,374
40	72,900,654
41	73,612,433
42	73,792,445
43	72,890,644
44	73,400,767
45	72,868,525
46	73,250,280
47	72,673,963
48	72,982,134
49	74,157,626

Sum	4,357,414,194				
Mean	88,926,820	9,790,900	1,459,075	11,249,975	6,282,800
SD	20,852,571.60		283,338.51	283,338.51	5,056,096
SE	2,978,938.80		141,669.25	141,669.25	3,575,200
			Room charge (Total)	-	-
				11,249,975	6,282,800

Adjust cost of PD complication with rate of PD complication in parameter sheet

**Room charge**	
RSCM room charge class II = 325,000	325000
RSKG room charge class II = 225,000	225000
Mean room charge class II	**275000**
	50000
No of LOS for PD initial	
LOS-PD-Initial	9
No of LOS for Peritonitis	
LOS-PD-peritonitis	7

Total direct cost		SE
Total PD initial	13,724,975	141,669.25
Total PD routine	88,926,820	2,978,938.80
Total PD complicatior	8,207,800	3,575,200

HD

Routime Costs included routine HD dialysis and other comorbidities

No	Direct Med Cost for Routine HD per visit from Hospital billing	Direct Med Cost for Routine HD per Year	Initial HD	LOS	HD Complication
1	1,156,205	120,245,324			
2	1,481,695	154,096,263			
3					
4	877,626	91,273,062			
5	1,049,104	109,106,782			
6	1,195,928	124,376,503			
7					
8	1,140,140	118,574,574			
9					
10	1,040,627	108,225,232			
11	1,129,980	117,517,944			
12	1,042,041	108,372,244			
13	1,151,391	119,744,692			
14					
15	1,495,271	155,508,190			
16	1,070,554	111,337,592			
17	1,028,543	106,968,504			34,463,124
18	1,405,648	146,187,342			
19	941,585	97,924,888			
20	1,053,896	109,605,145			
21	1,120,907	116,574,367			
22	1,126,771	117,184,148			
23	1,438,436	149,597,331			
24	1,355,379	140,959,398			
25	1,107,005	115,128,553			10,920,760
26	1,036,775	107,824,571			
27	1,060,041	110,244,224			
28	862,434	89,693,121			
29	1,094,239	113,800,886			24,761,975
30	872,630	90,753,547			
31	1,077,047	112,012,842			
32	939,744	97,733,400			14,473,000
33	928,403	96,553,913			680,400
34	880,970	91,620,852			8,281,800
35	965,641	100,426,655	8,803,625	10	4,155,825
36	834,410	86,778,640			5,439,450

	37	1,193,443	124,118,121			
	38	912,854	94,936,848	9,331,200	8	
	39	975,403	101,441,893			
	40	1,011,767	105,223,733			
	41	1,075,753	111,878,361			
	42	927,382	96,447,706	15,358,500	11	
	43	873,650	90,859,600			2,265,875
	44	946,825	98,469,800			
	45	921,323	95,817,589			
	46	1,046,722	108,859,133			
	47					
	48	943,953	98,171,163			3,226,175
	49	937,842	97,535,600			
	50					
	51	1,042,347	108,404,053			3,801,575
Sum		47,770,330	4,968,114,333	33,493,325	29	112,469,959
Mean		1,061,563	110,402,541	11,164,442	10	10,224,542
SD		164,200.52	17,076,853.97	3,641,727	2	10,609,162
SE		24,477.57	2,545,667.09	2,102,552	0.88	6,125,203

No of HD visit per year (expert opinion)

OPD visits	104	

No of LOS for HD initial

LOS-HD-initail	9.67	1

No of LOD for HD Complication

LOS-HD-Comp	9.82	1.86

Room Charge

RSCM room charge class II = 325,000

RSKG room charge class II = 225,000

Mean room charge cl	**275000**

Total direct cost		**SE**	
Total HD initial cost	13,822,775	2,102,552	
Total HD Routine	110,402,541	2,545,667	
Total HD complicatioı	12,924,542	6,125,203	Adjust cost of HD complication with rate of HD complication in parameter sheet

LOS

16

12

19

14
2
10
5
11

5

9

5

9.82
5
2
