# Supplementary material for: Economic evaluation of policy options for dialysis in end-stage renal disease patients under the universal health coverage in Indonesia
Source: PLoS One. 2017 May 18;12(5):e0177436. doi: 10.1371/journal.pone.0177436 (PMC5436694; doi:10.1371/journal.pone.0177436)
Supplement: S2 File — (PDF) [file pone.0177436.s002.pdf]

PD	Family/Companion Income Loss in a month	Family/Companion Income Loss in YEAR
1	250000	3000000
2	150000	1800000
3	60000	720000
4	125000	1500000
5	225000	2700000
6	0	0
7	40000	480000
8	250000	3000000
9	70000	840000
10	300000	3600000
11	110000	1320000
12	109750	1317000
13	250000	3000000
14	150000	1800000
15	90000	1080000
16	120000	1440000
17	0	0
18	0	0
19	62500	750000
20	430000	5160000
21	150000	1800000
22	100000	1200000
23	300000	3600000

Indirect cost of PD	
Mean	1,743,783
SD	1,302,865
SE	271,666

12

HD	Family/Companion Income Loss in a month	Family/Companion Income Loss in YEAR
1	0	0
2	0	0
3	3000000	36000000
4	0	0
5	0	0
6	0	0
7	0	0
8	0	0
9	0	0
10	0	0
11	0	0
12	0	0
13	2800000	33600000
14	0	0
15	2400000	28800000
16	0	0
17	600000	7200000
18	700000	8400000
19	700000	8400000
20	0	0
21	0	0
22	0	0
23	0	0
24	0	0
25	240000	2880000
26	612000	7344000
27	0	0
28	200000	2400000
29	0	0
30	0	0
31	0	0
32	200000	2400000
33	300000	3600000
34	0	0
35	0	0
36	0	0
37	200000	2400000
38	0	0
39	0	0
40	0	0
41	0	0
42	0	0
43	1200000	14400000
44	0	0
45	0	0
46	0	0
47	0	0
48	0	0
49	0	0
50	0	0

Indirect cost of HD	
Mean	3,156,480
SD	8,061,043
SE	1,140,004
