# Supplementary material for: Economic evaluation of policy options for dialysis in end-stage renal disease patients under the universal health coverage in Indonesia
Source: PLoS One. 2017 May 18;12(5):e0177436. doi: 10.1371/journal.pone.0177436 (PMC5436694; doi:10.1371/journal.pone.0177436)
Supplement: S3 File — (PDF) [file pone.0177436.s003.pdf]

**Total entry data**	**68**
**CAPD**	**41**
**HD**	**27**

Age group	n
< 20 y.o.	0
20 - 30	8
31 - 40	17
41 - 50	15
51 - 60	14
61 - 70	9
> 70 y.o.	5

Gender	n
Male	38
Female	30

Education level	n
No schooling	1
Primary School	13
Junior High School	5
High School	25
College or above	24

Initial Therapy	n
CAPD	3
HD	65

Current Therapy	n
CAPD	41
HD	27

Duration of Therapy	n	
< 12 months	12	
12 -24 months	29	
> 24 months	25	
missing	2	

Comorbidities	n	Total = 68
None	3	
Yes	64	
Missing	1	

Number of Comorbidities	n	Total = 68
One	30	
Two	35	
More than two	2	
Missing	1	

Type of Comorbidities	n	Total =56
Diabetes Mellitus (DM)	1	
Hypertension (HT)	25	
DM and HT	11	
Others	19	

*Others = Hepatitis, SLE, hyperuricemia, asthma, lupus, gastritis

History of Hospital Admission	n
Yes	31
No	37

*Reason: infection (incl. peritonitis), high blood pressure, anemia, dyspneu, bleeding, hypoalbuminemia, etc

Av. Duration of Hospital Admission **7.96 days**
(2 - 60 days)

Companion to Hospital	n
Yes	53
No	14
Missing	1

Companion	n
Family	51
Other than family	2

Av. Number of companion	0,913 = 1 person
(1 - 3 person)	

Time spent in the hospital	
Av. CAPD patient	182 mins
Av. HD patient	313 mins

Transportation	
Private transport	40
Public transport	26
Combined	2

**Av. Transportation time**	**85 mins**	
**Employment (patient) - total**	**n**	
Employed	23	
Unemployed	45	
Missing	1	
**Employment - CAPD**	**n**	
Employed	13	
Unemployed	28	
**Employment - HD**	**n**	
Employed	10	
Unemployed	17	
**Type of Work - Patient**	**n**	
Full time	9	
Part time	4	
Enterpreneur	10	
**Av. days leaving work to hospital - patients**	**n**	
Total patients	3.347	(1-12 days)
CAPD patient	1.46	(1-2 days)
HD patient	5.8	(5-12 days)
**Av. Salary - Patient**	**n**	
Total	3,642,658	(200,000 - 14,500,000)
CAPD patient	4,194,872	(200,000 - 14,500,000)
HD patient	2,924,780	(200,000 - 5,107,800)
**Employment (companion)**	**n**	
Employed	31	
Unemployed	18	
Missing	2	
**Type of Work - Companion**	**n**	
Full time	17	
Part time	4	
Enterpreneur	9	
*Working for patient as driver	1	

Av. Days leaving work to hospital - companion	n	
Total	2.23 days	(1-8 days)
CAPD	1.2 days	(0.33-8 days)
HD	3.91 days	(1-8 days)

Av. Salary - Companion	n	
Total	3,031,775	(300,000 - 20,000,000)
CAPD	3,127,250	(300,000 - 20,000,000)
HD	2,858,182	(500,000 - 4,200,000)

**Sessions of PD per day**	**4**	
**Dialysis sessions per patient per week**	**2**	

Complications	n	
CAPD	18	*infection, peritonitis, bleeding, anemia, dyspneu, hernia
HD	14	*dyspneu, hypoglicemia, anaphylactic reaction, oedema

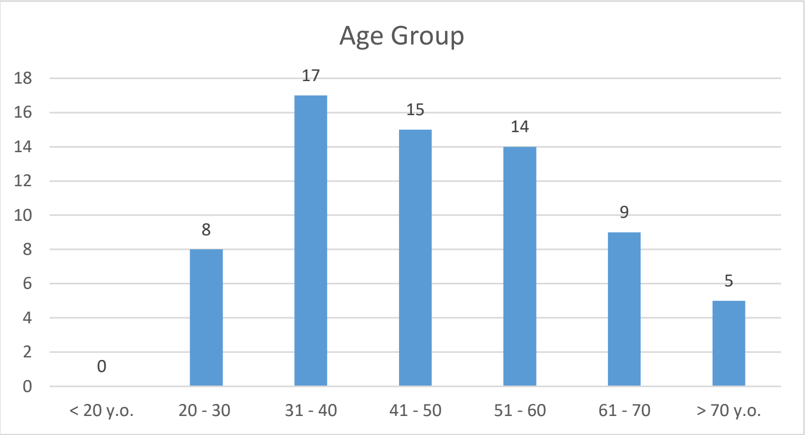

Age Group
18
16
14
12
10
8
6
4
2
0
0
8
17
15
14
9
5
< 20 y.o.
20 - 30
31 - 40
41 - 50
51 - 60
61 - 70
> 70 y.o.

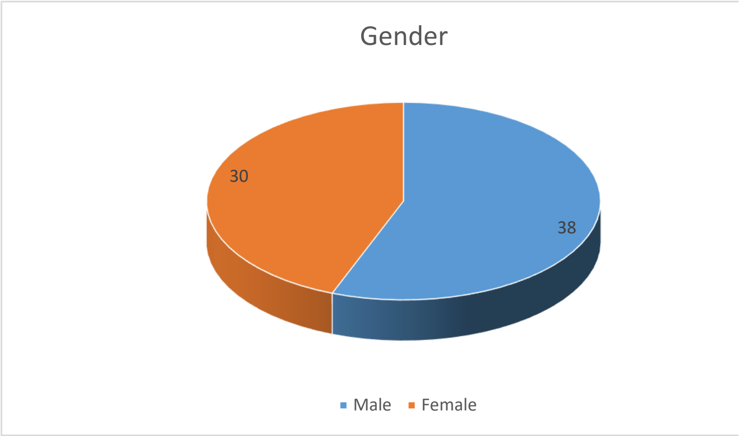

Gender
30
38
Male
Female

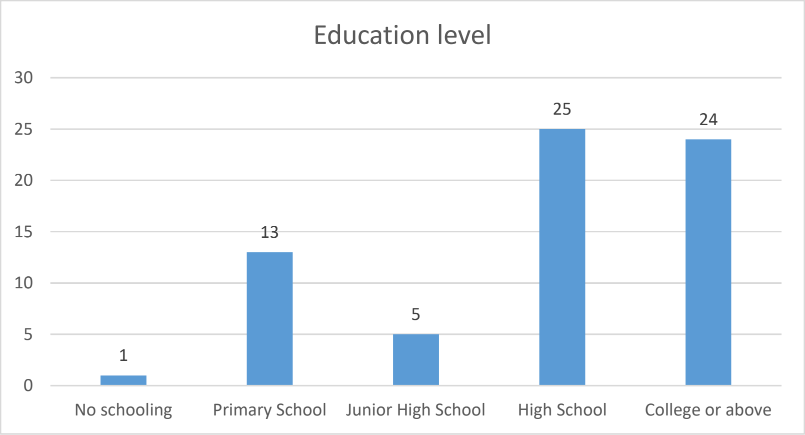

Education level
30
25
20
15
10
5
0
1
13
5
25
24
No schooling
Primary School
Junior High School
High School
College or above

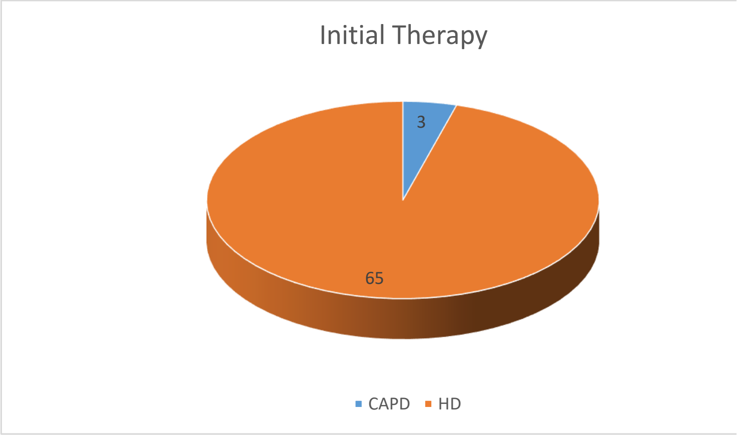

Initial Therapy
3
65
CAPD
HD

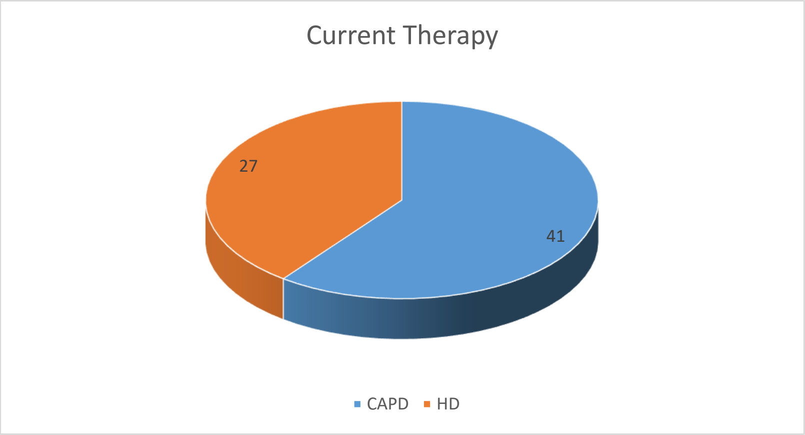
Current Therapy
27
41
CAPD
HD

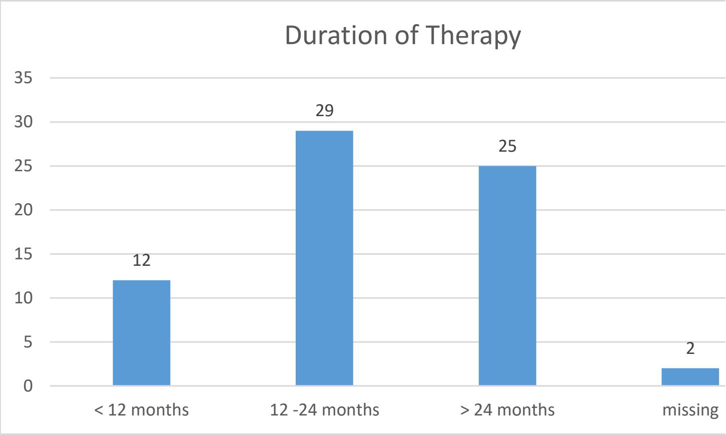
Duration of Therapy
35
30
25
20
15
10
5
0
12
29
25
2
< 12 months
12 -24 months
> 24 months
missing

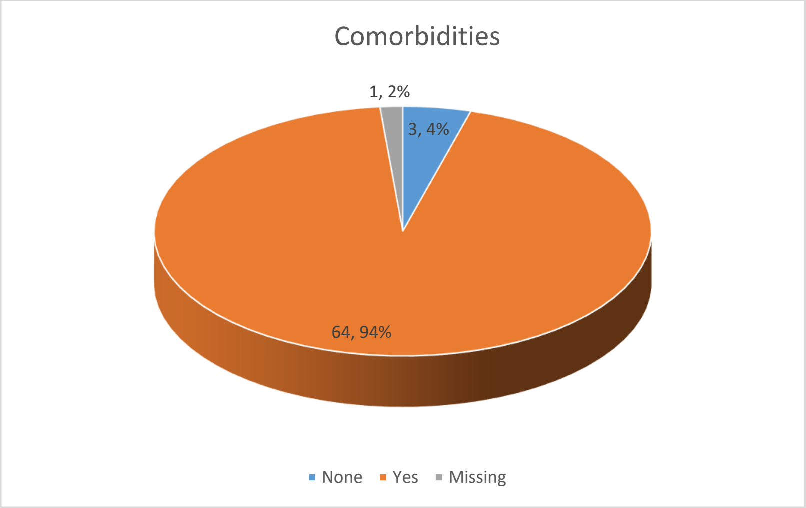
Comorbidities
1, 2%
3, 4%
64, 94%
None
Yes
Missing

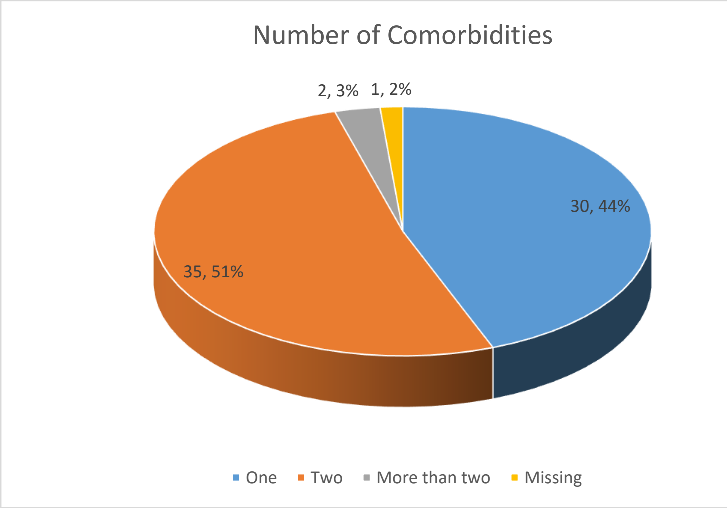
Number of Comorbidities
2, 3%
1, 2%
30, 44%
35, 51%
One
Two
More than two
Missing

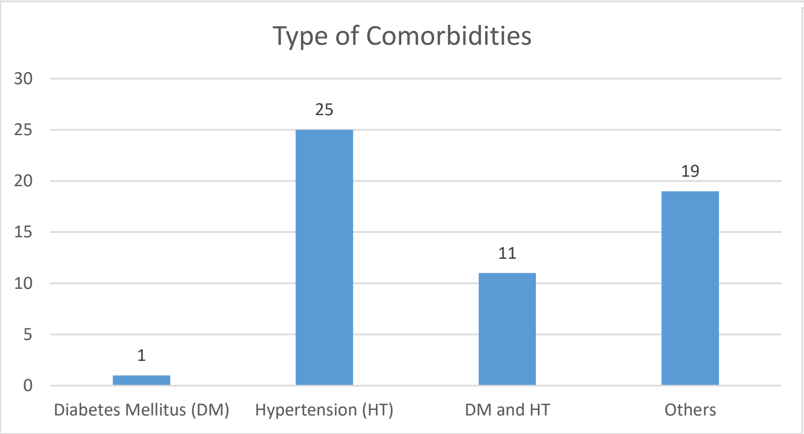
Type of Comorbidities
30
25
20
15
10
5
0
1
25
11
19
Diabetes Mellitus (DM)
Hypertension (HT)
DM and HT
Others

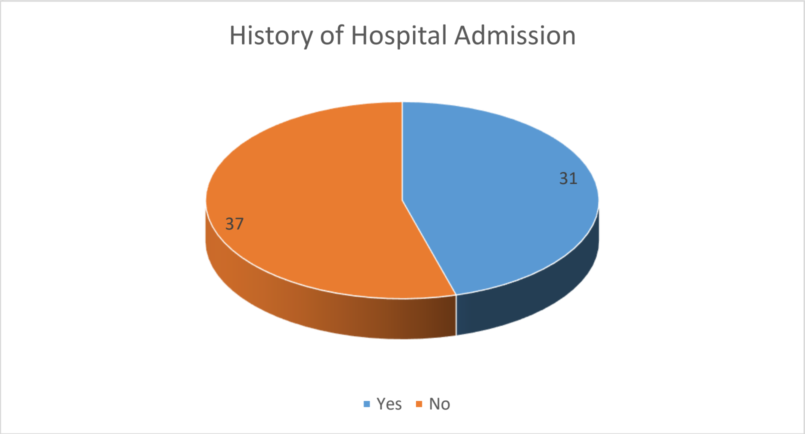
History of Hospital Admission
31
37
Yes
No

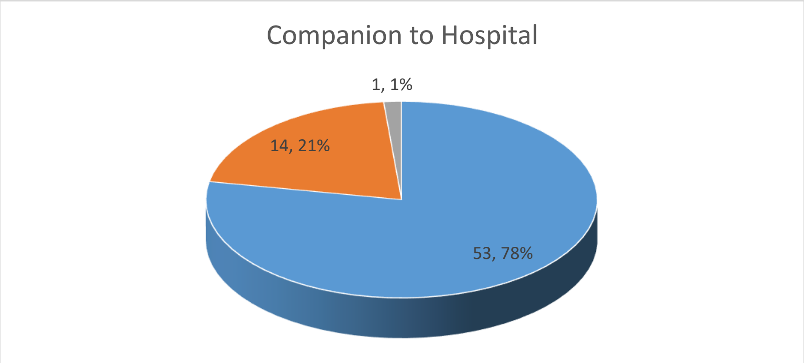
Companion to Hospital
1, 1%
14, 21%
53, 78%

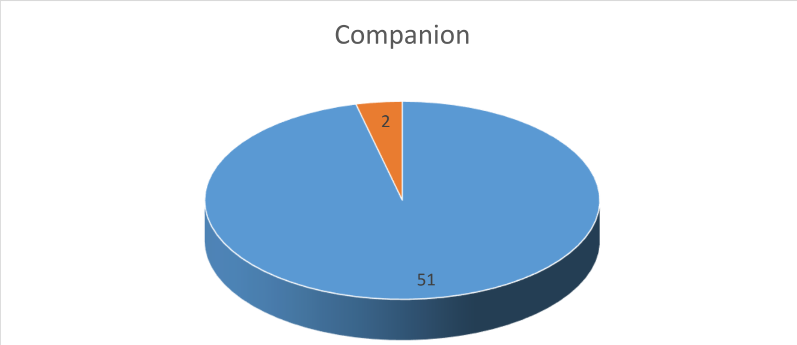
Companion
2
51

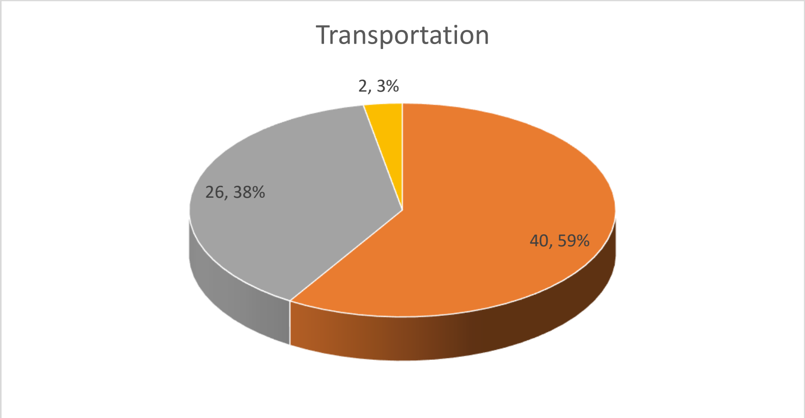
Transportation
2, 3%
26, 38%
40, 59%

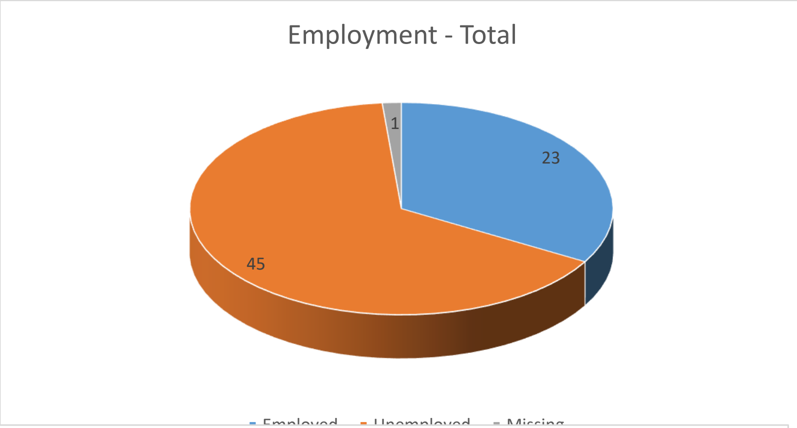
Employment - Total
1
23
45

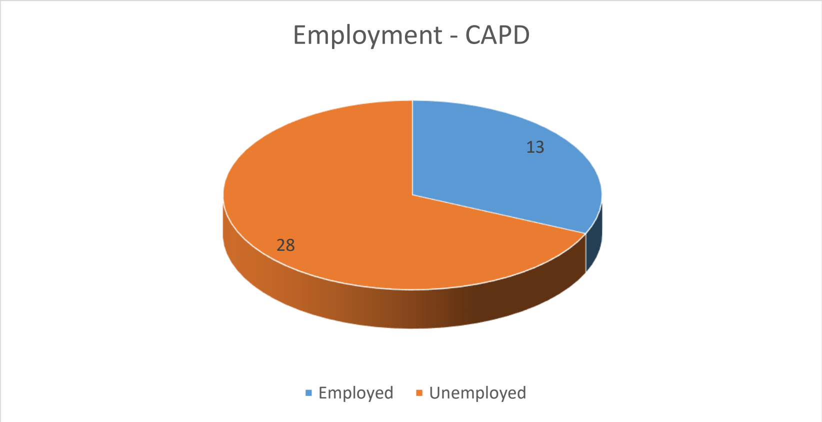
Employment - CAPD
13
28
Employed
Unemployed

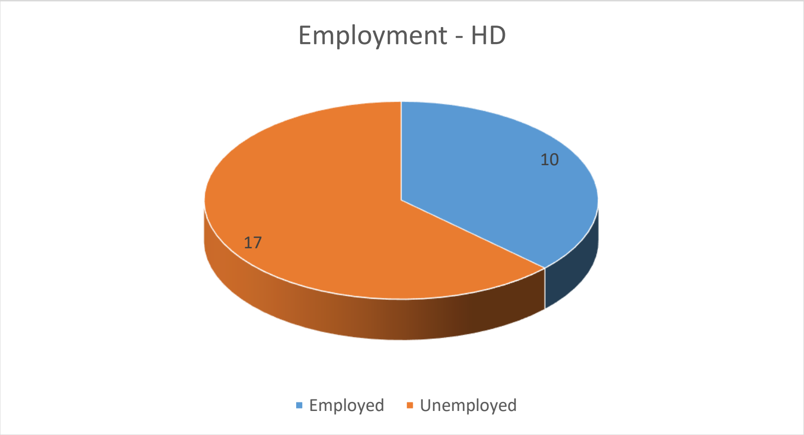
Employment - HD
10
17
Employed
Unemployed

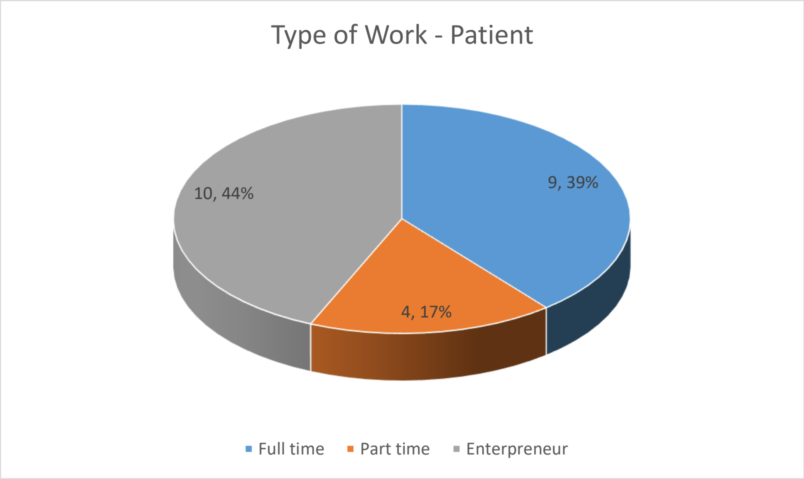
Type of Work - Patient
9, 39%
4, 17%
10, 44%
Full time
Part time
Enterpreneur

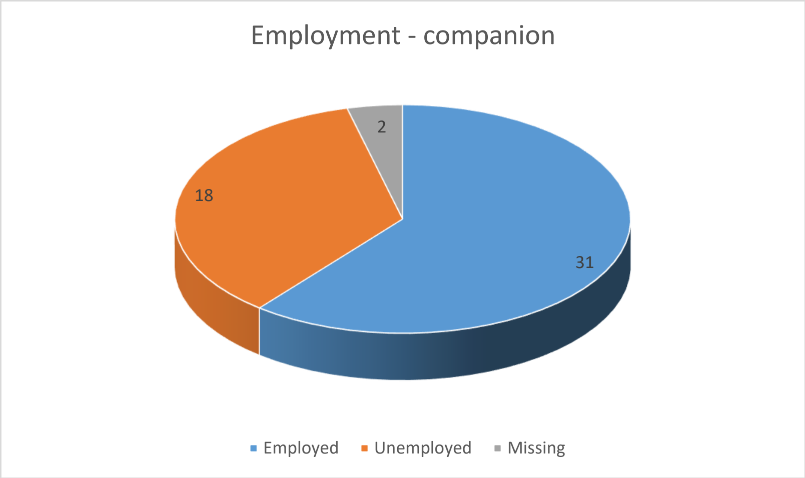
Employment - companion
2
18
31
Employed
Unemployed
Missing

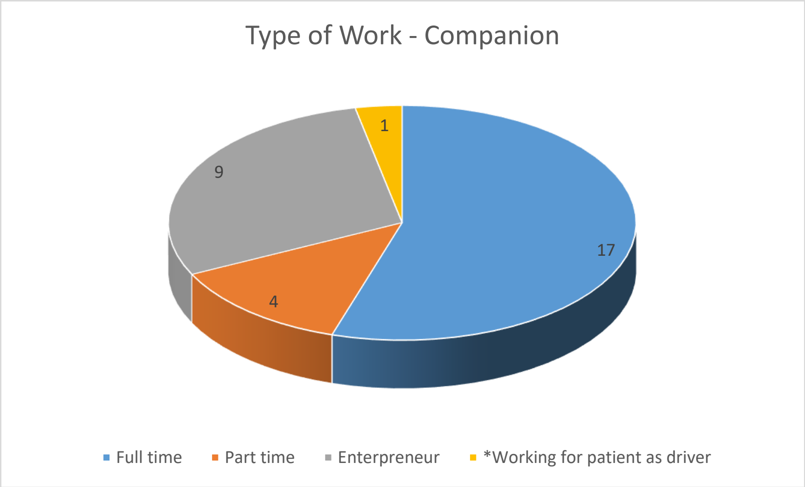
Type of Work - Companion
1
9
17
4
Full time
Part time
Enterpreneur
*Working for patient as driver

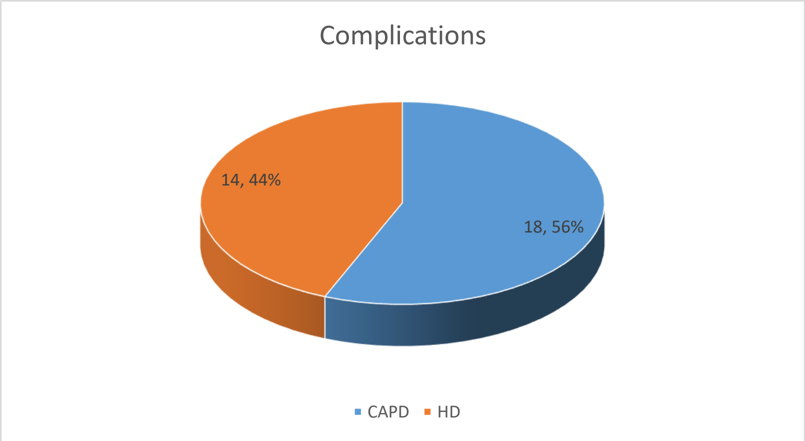
Complications
14, 44%
18, 56%
CAPD
HD
